# Intraventicular Delivery of IL‐27 Gene Exerts Neuroprotection and Ameliorates Cognitive Deficits in A Mouse Model for Alzheimer's Disease

**DOI:** 10.1002/alz70855_100804

**Published:** 2025-12-23

**Authors:** Zichun Xiao

**Affiliations:** ^1^ Tianjin Medical University General Hospital, Tianjin, China; Tianjin Neurological Institute, Tianjin, China

## Abstract

**Background:**

Alzheimer's disease (AD) is a major challenge for the global public health system. AD is the most common (60‐70%) type of dementia. Understanding the pathogenesis of AD is vital in developing effective, therapeutic or preventive strategies. The successful completion of a few late phase clinical trials and approval of monoclonal antibodies against amyloid protein in recent years reinforced the amyloid cascade hypothesis (ACH). However, the marginal efficacies of anti‐amyloid therapy and its high frequencies of side effects (e.g: ARIA‐E and AIRA‐H) indicate other major player(s) in the pathogenesis of AD and warrant more effective, safer therapies.

**Method:**

In the current study, we generated recombinant Adeno‐Associated Virus (rAAV) vector expressing mouse IL‐27 (AAV‐IL‐27), enhanced green fluorescent protein (AAV‐EGFP) and Control vector (AAC‐Ctrl) without IL‐27 cDNA. AAV‐IL‐27, AAV‐EGFP or AAV‐Ctrl were delivered by stereotactic injections into the lateral ventricles of Alzheimer's disease 5xFAD mice. Mice were monitored and evaluated for various durations after a single delivery of treatment, from 4 to 7.5 months.

**Result:**

Using flow cytometry and immunohistochemistry staining, we showed that intraventricular delivery of AAV‐IL‐27 successfully transduced neurons () with high levels of IL‐27 expression. Meanwhile, enhanced expression of IL‐27R were evident in regions such as the hippocampus and cerebral cortex. 5xFAD mice treated with AAV‐IL‐27 had preserved neuro‐cognitive function as revealed by neurobehavioral tests. Furthermore, AAV‐IL‐27 treatment was found to reduce the expression of pro‐inflammatory cytokine IL‐1 and limit neuronal synaptic damage.

**Conclusion:**

Together with our previous reported findings, data from the current study suggest an intrinsic, neuroprotective pathway of IL‐27 that alleviates neuronal damage associated with AD via modulation of the inflammatory responses within the CNS.

